# Old and New Insights in the Treatment of Thyroid Carcinoma

**DOI:** 10.4061/2010/279468

**Published:** 2010-04-13

**Authors:** Joan Manel Gasent Blesa, Enrique Grande Pulido, Mariano Provencio Pulla, Vicente Alberola Candel, Juan Bautista Laforga Canales, Miguel Grimalt Arrom, Patricia Martin Rico

**Affiliations:** ^1^Departament d'Oncologia Mèdica, Hospital de Dénia, Marina Salud, Partida de Beniadlà s/n, Dénia, Alacant, Spain; ^2^Departamento de Oncología Médica, Hospital Ramón y Cajal, Ctra. de Colmenar Viejo km 9100, 28034 Madrid, Spain; ^3^Departamento de Oncología Médica, Hospital Puerta de Hierro, Calle Manuel de Falla N^*∘*^ 1 del Municipio de Majadahonda, 28222 de Madrid, Spain; ^4^Departamento de Oncología Médica, Hospital Arnau de Vilanova, Calle de La Marina Alta s/n, 46015 Valencia, Spain; ^5^Departament d'Anatomia Patològica, Hospital de Dénia, Marina Salud, Partida de Beniadlà s/n, Dénia, Alacant, Spain; ^6^Departamento de Cirugía Oncológica Benidrom, Hospital de Levante, Calle del Doctor Santiago Ramón y Cajal, 7, 03503 BenidormAlacant, Spain; ^7^Departament de Medicina Interna, Hospital de Dénia, Marina Salud, Partida de Beniadlà s/n, Dénia, Alacant, Spain

## Abstract

Thyroid cancer is the endocrine tumor that bears the highest incidence with 33 550 new cases per year. It bears an excellent prognosis with a mortality of 1530 patients per year (Jemal et al.; 2007). We have been treating patients with thyroid carcinoma during many years without many innovations. Recently, we have assisted to the development of new agents for the treatment of this disease with unexpected good results. Here we present a review with the old and new methods for the treatment of this disease.

## 1. Introduction

Thyroid carcinoma usually presents as a palpable thyroid nodule or a nodule that is discovered incidentally on neck imaging for evaluation because of another reason. Less commonly it may present as a lateral neck mass [1]. The rate of malignancy for a thyroid nodule is of only 4-5% [2, 3]. The prevalence of thyroid nodules increases with age and is estimated to occur in roughly 50% of the US population 50 years of age and older [4]. Thyroid cancer may present as a dominant nodule in a multinodular gland or as a solitary nodule. The risk per gland is equivalent in multinodular and in solitary nodule [5]. Incidentally discovered nodules carry a similar risk of malignancy [6]. Every nodule should be evaluated. Upon initial presentation, a careful history is essential because it can reveal information that immediately stratifies a patient into a high risk category. High risk factors include a prior history of head and neck radiation including radiation for Hodgkin's disease [7], radiation exposure from the Chernobyl accident [8] and a family history of thyroid cancer. The risk of thyroid cancer in first degree relatives of patients with differentiated thyroid cancer has been shown to be six times that of the general population [9]. Other important high risk factors include a history of rapid growth of a solid nodule, pain, dysphagia, or dysphonia. Although less than 20% of patients with thyroid carcinoma will present with a regional metastasis, a neck mass in the presence of a thyroid nodule raises the likelihood of malignancy. Young age (<20 years), older age (>70 years), and male gender may also represent an increased risk [10]. To most accurately determine the risk of malignancy, it is essential to consider a variety of factors (Table 1). There is no single laboratory study that is able to determine the presence of thyroid malignancy. Although elevated baseline calcitonin levels represent occult medullary carcinoma in 10–40% of cases, the presence of false positive elevations, the low incidence of medullary carcinoma, and the high cost of testing have led to recommendations against routine testing in the United States [11]. Calcitonin levels are usually reserved for high risk patients and those with a family history of medullary thyroid carcinoma or multiple endocrine neoplasia (MEN). Thyroglobulin is not useful in the work up because there are a variety of conditions that can lead to elevated levels including thyroid inflammation, glandular stimulation or injury following radiation, surgery, or even a biopsy. TSH should be measured to exclude a toxic nodule, as toxic nodules are almost never malignant and generally do not require fine needle aspiration. If the TSH is suppressed, then radioisotope imaging should be performed to confirm and localize the toxic nodule(s).

### 1.1. Clinical Staging and Risk Group Classification

Clinical staging and risk group classification is particularly important in the management of thyroid cancer because it often will direct management, in particular the decision to treat the patient with radioactive iodine, external beam radiation, or combination therapy. The lack of prospective randomized studies assessing the relationships between tumor stage, treatment, and outcome, means that the majority of data that is used to create risk-group classifications is derived from retrospective reviews. As a result, there are variety of classification systems that are based on factors such as age, tumor size, gender, tumor grade, multicentricity, metastatic disease, and other variables. The AGES system [12] is based on factors including age, grade, extent, and size of the tumor (Table 2). In the AGES system, those patients with an aggregate score 4+ are high risk and those with a score less than 4 are low risk. The AMES system [13] considers age, distant metastasis, extent, and size of tumor (Table 3). Additionally, there are systems devised by the European Organization for Research on Treatment of Cancer (EORTC) [14], the National Thyroid Cancer Treatment Cooperative Study [15], and others [13, 16]. Most of these classification systems utilize similar information with minor variations. Irrespective of the classification system that is used, it is important to consider risk group stratification during the management of a patient with thyroid cancer. Separate from these stratification systems is the TNM tumor staging system (Table 4) which serves to provide a uniform language when evaluating management and outcome.

## 2. The Old Insights

### 2.1. Papillary Thyroid Carcinoma

Papillary thyroid carcinoma (PTC) is the most common form of the follicular cell derived carcinomas and comprises three quarters of all newly diagnosed thyroid cancers [17]. PTC is derived from the follicular cells. These cells tend to concentrate iodine and secrete thyroglobulin. As a result, surveillance and detection of recurrence can be relatively straightforward. The prognosis for PTC is usually excellent. 

Papillary thyroid cancers usually carry mutations and rearrangements that activates the mitogen activated protein kinase (MAPK). Rearrangements of RET and NTRK1 tyrosine kinases, activating mutations of BRAF and activating mutations of RAS are sequential components of the MAPK activation [18]. Papillary thyroid cancers are also connected with rearrangements of RET and NTRK1 [19]. These rearrangements result in the production of chimeric proteins, named as RET/PTC and TRK. Any these genes is expressed in normal thyroid epithelial cells. Consequently, patients with MEN2 syndromes do not have an increased incidence of papillary thyroid cancer. In adults, close to 40 percent of sporadic papillary cancers have these rearrangements, with those in RET being about three times more common [20]. The incidence of RET rearrangements in papillary cancers is higher in children [21], affecting up the 80 percent in the case of children exposed to external x-irradiation and those exposed to the Chernobyl nuclear accident. 

#### 2.1.1. The Case of the BRAF and RAS Mutations

The BRAF proteins are serine-threonine kinases that activate the RAF/MEK/MAPK signaling pathway. The T1799A mutation of the BRAF gene, affects up to 69 percent of papillary thyroid cancers [18, 22–24]. In one report, BRAF mutations were found in 44 percent of 500 patients with papillary thyroid cancer [25]. BRAF mutations may be associated with a worse clinical prognosis [26]. BRAF mutations are associated with extrathyroidal tissue invasion, lymph node metastases and advanced tumor stage. Surprisingly it has been described acquird BRAF mutations in lymph node metastases [27].

BRAF point mutations are less frequent in childhood thyroid cancers, including those children affected by the Chernobyl nuclear calamity [28]. *RAS mutations*. Although activating ras mutations are found in follicular adenomas, they have also been reported in follicular variant of papillary thyroid cancers [29]. 

There may be an increased incidence of papillary cancer in patients with familial adenomatous polyposis [30]. The papillary thyroid cancers of these group patients with familial adenomatous polyposis have frequent rearrangements of RET [31]. 

Papillary thyroid cancers can occur as a familial syndrome [32]. Other family studies have indicated a familial predisposition at 2q21 to PTC, as well as a familial predisposition at 8q24 to PTC with melanoma [33].

#### 2.1.2. Management of Papillary Thyroid Carcinoma

Surgery is the primary treatment for papillary thyroid cancer and a complete resection offers the best chance of cure [10, 34]. While the role of a complete surgical resection is established, the extent of the initial surgical resection has been topic of controversy. Primary management of thyroid malignancy has included nodulectomy, hemithyroidectomy with and without isthmusectomy, subtotal thyroidectomy, and total thyroidectomy. The high rate of recurrence associated with nodulectomy has confirmed that this approach is inadequate. While there has been a trend toward more aggressive surgical resection based on the well-documented multicentric nature of the disease, there are still some who insist that a partial thyroidectomy is sufficient [35] Proponents of total thyroidectomy point to data that demonstrates a rate of multicentric bilateral disease ranges from 18% to 46% [36, 37]. Opponents of total thyroidectomy suggest that multicentric bilateral disease is often microscopic and not clinically significant. This is further supported by the observation that several large studies have failed to demonstrate a survival advantage to total thyroidectomy when compared with hemithyroidectomy [38, 39]. In spite of these findings, most clinicians agree that there is a subset of patients who benefit from total thyroidectomy while there is another distinct subset of patients that may be treated more conservatively. The concern regarding “over-treating” a patient by performing a total thyroidectomy instead of a hemithyroidectomy relates to the surgical risk including an increased risk of vocal cord paralysis and a risk of permanent hypoparathyroidism. In an effort to guide surgical management, the AGES and the AMES classification systems represent two commonly used systems that are used to classify patients into either a “high risk” group or a “low risk” group. It has been suggested that “low risk” patients can be managed with a thyroid lobectomy and isthmusectomy while “high risk” patients are better managed with a total thyroidectomy. Most surgeons agree with the recommendation to perform a total thyroidectomy when faced with a high risk patient. However the controversy exists in the low risk patient subgroup. Several studies have failed to demonstrate that low risk patients experience an improvement in cause-specific mortality after undergoing a total thyroidectomy [12, 40] while several large studies have demonstrated that there is a benefit to total thyroidectomy [39, 41]. The conflicting data can make the decision-making process a difficult one. As a result, not all surgeons favor such guidelines and many prefer to perform a total thyroidectomy in spite of the risk stratification suggesting that the rate of morbidity in the hands of an experienced surgeon is exceedingly low [42]. A recent study found that surgeons who perform more than 100 thyroidectomies per year reported complication rates of 4.3% while those surgeons who performed less than 10 thyroidectomies per year reported a four-fold increase in complication rate. In spite of the complication rate, when a subtotal thyroidectomy is performed for the management of thyroid carcinoma, it becomes difficult to follow thyroglobulin levels for surveillance [43]. This may represent the most compelling argument to perform a total thyroidectomy for malignancy. Finally, the American Thyroid Association (ATA) recently recommended that a total thyroidectomy should be performed for the management of well-differentiated thyroid carcinoma [20].

Whereas one recent expert consensus guideline from the ATA [4, 44], recommended consideration of routine central neck dissection for most patients with papillary thyroid cancer, the guideline from the National Comprehensive Cancer Network only recommends central neck dissection in the presence of grossly positive metastasis.

### 2.2. Follicular Thyroid Carcinoma

Follicular carcinoma is less common than papillary thyroid carcinoma [17] and typically behaves more aggressively than papillary thyroid carcinoma. The aggressive nature of follicular thyroid carcinoma is largely a result of its propensity for hematogenous dissemination. Unlike papillary thyroid carcinoma, which spreads through lymphatics, follicular carcinoma has a tendency to metastasize hematogenously to the lung and bones. The incidence of distant metastasis at presentation is not uncommon occurring between 10 and 20% of new cases [45–47]. As a result, a bony lesion or pathologic fracture may represent the initial presentation. The key to effective management of follicular carcinoma is making an early diagnosis and total thyroidectomy. 

*The genetics aberrations of the Follicular Thyroid cancer* includes the translocation (t[2,3][q13;p25]) [48] that results in fusion of part of the DNA-binding segment of the PAX8 gene and the peroxisome proliferator-activated receptor gamma 1 (PPAR-gamma-1) gene; PAX8 is a thyroid transcription factor and PPAR-gamma-1 is a transcription factor that stimulates cell differentiation and inhibits cell growth. Its presence, may be useful to distinguish PTC from Follicular Thyroid Cancer. 

Overexpression of normal c-myc and c-fos genes, as well as mutations of H-ras, N-ras, and K-ras protooncogenes, is found in follicular adenomas, follicular cancers, and occasionally papillary cancers [49, 50]. Allelic losses in these genes, also occur frequently in follicular cancers [51]. 

Hypermethylation of RASSF1A, a known tumor suppressor gene, has been described in one study, in 9 of 12 follicular thyroid cancers and in 44% of benign adenomas, and in 20% of papillary thyroid cancers reflecting and early step in thyroid carcinogenesis [52].

#### 2.2.1. Management of Follicular Thyroid Carcinoma

In contrast to papillary thyroid cancer, the frozen section pathological analysis is not helpful in making the diagnosis of follicular carcinoma because capsular and vascular invasion must be identified and this requires fine section analysis that cannot be completed with FNA or at the time of frozen section [53, 54]. The definitive diagnosis of follicular thyroid carcinoma can be made only on permanent section. Once the diagnosis of follicular carcinoma has been confirmed, the decision to proceed with a completion thyroidectomy or follow the patient is based on a variety of factors including risk group analysis. Most agree that high risk patients require a total thyroidectomy. Not only does a total thyroidectomy improve prognosis but it is very helpful in achieving accurate surveillance by facilitating RAI administration and thyroglobulin monitoring. There is controversy regarding the low risk group patients who present with minimally invasive lesions. The decision to perform a completion thyroidectomy is often based on age of the patient and invasiveness of the tumor. In patients greater than 50 years old or those with extensive invasion, we recommend a completion thyroidectomy. 

Patients with low grade tumors, tumors that demonstrate minimal invasion, can be managed conservatively with observation and serial ultrasound examination. Minimally invasive follicular thyroid carcinoma tends to behave like a follicular adenoma. There is a very low incidence of metastasis in low grade follicular carcinoma [55, 56] and unlike papillary thyroid carcinoma, minimally invasive lesions are rarely multifocal. However, as follicular lesions grow larger than 3 cm, nearly 30% will demonstrate malignant conversion [57]. While some have advocated suppressive therapy for low risk patients, this approach has failed to demonstrate the benefit of suppressive therapy.

#### 2.2.2. Management of Regional Disease in Well Differentiated Thyroid Carcinoma

Unlike squamous cell carcinoma, the impact of lymph node metastasis from thyroid cancer on survival is negligible, at least in the younger population. Several studies have suggested that lymph node metastasis may not impact survival [58, 59] however when adjusted for age, it is clear that although regional disease does not significantly impact on survival in young patients, it does decrease survival in patients over the age of 45 [60]. Long term studies have confirmed these observations and identified that in the population of patients over 45 years old, regional metastasis does impact survival [37]. As a result, most experts agree that clinically involved regional lymph nodes should be managed with a lymph node dissection. There is, however, controversy regarding the impact of elective neck dissection on survival. This is largely a result of the paucity of controlled randomized studies. Thyroid cancer commonly progresses in a defined pathway from the first echelon lymph nodes of the paratracheal region to the lateral compartment (levels III and IV), however it has been documented that regional disease may occur in the jugular chain without evidence of disease in the paratracheal basin in as many as 18% of cases [59]. In spite of this rather high rate of “skip metastasis”, most surgeons do not advocate a lateral neck dissection unless there is clinical evidence of disease in the lateral compartment. The rate of occult regional metastasis in papillary thyroid carcinoma is relatively high however occult metastasis in follicular carcinoma is rare. Hence, in patients with follicular carcinoma, an elective lymph node dissection is not commonly performed in the N0 neck. If lymphadenopathy is detected, a paratracheal and pretracheal central compartment neck dissection should be performed. In papillary thyroid carcinoma, the high rate of paratracheal disease constitutes an ipsilateral paratracheal neck dissection when there is clinical evidence of disease. Node plucking is not acceptable as the rate likelihood of recurrence is exceedingly high [61].

#### 2.2.3. Management of the N0 Neck

The approach to management of the N0 neck in papillary carcinoma has been a topic of controversy largely because there are few randomized, controlled, long term studies that demonstrate the impact of surgical treatment of the N0 neck. Currently, most surgeons perform a pretracheal and ipsilateral paratracheal lymph node dissection on high risk patients (those patients aged 45 years or older, or patients with tumors greater than 4 cm, or invasive disease). Only when the thyroid tumor crosses the midline is a contralateral lymph node dissection warranted. When a contralateral lymph node dissection is performed, the patient must be informed of the increased risk related to transient and permanent hypoparathyroidism [62]. As discussed earlier, in spite of the relatively high rate of documented skip metastasis, most surgeons do not advocate a lateral neck dissection. The rationale for this approach stems from the lack of data demonstrating the benefit of a lateral neck dissection for subclinical disease and the prevailing belief that I131 can be used to manage microscopic lateral metastasis.

#### 2.2.4. Management of N+ Neck

Unlike follicular carcinoma in which lymph nodes metastases are rare, metastasis in papillary thyroid carcinoma is common. While only a small proportion of patients will initially present with a neck mass [63], a significant number of patients will harbor subclinical paratracheal disease at the time of diagnosis. The size of the primary thyroid tumor has little bearing on the extent or location of regional disease. Several groups including our own, have found that while large cancers of the thyroid may remain localized, microcarcinoma may present with extensive regional disease [64]. As a result, it is difficult to predict the risk of regional disease based on the size of the tumor and therefore every patient must be evaluated for regional metastasis. 

When lymph node metastasis is identified, a neck dissection is warranted. I131 has not been demonstrated effective for management of gross regional disease [65]. In the past, there was controversy regarding the extent of the dissection however most surgeons now advocate a selective neck dissection (levels III, IV, and VI) in which the jugular vein, sternocleidomastoid muscle, and the accessory nerve are preserved. Although rare, when invasion of the surrounding structures occurs, a modified neck dissection may be warranted.

#### 2.2.5. Radioiodine Therapy

Remnant ablation with 131I is routinely performed in all patients except for very low risk patients after total thyroidectomy to decrease the risk of recurrence and facilitate monitoring of thyroglobulin. Usually thyroid replacement is discontinued and a low iodine diet instituted in an effort to increase the avidity of the residual thyroid tissue. Once the TSH has risen above 25 to 30 mU/L, the patient is given radioiodine in doses ranging 30 mCi to 100 mCi. Patients in whom residual microscopic disease is suspected and those with more aggressive tumor histology or known distant metastasis are administered higher doses of I131 in the range of 100 to 300 mCi. Younger patients with iodine avid tumors tend have a favorable response to 131I. 

Hypothyroidism can be avoided by administering recombinant human TSH (rhTSH, thyrotropin alfa) before administration of 131-I for remnant ablation [66, 67]. Short-term results of radioiodine ablation appears to be similar after rhTSH administration or thyroid hormone withdrawal. In one study the radiation dose to the thyroid bed of a tracer amount of radioiodine was similar after two injections of thyrotropin alfa or thyroid hormone withdrawal [68]. In retrospective studies, the percentage of patients with successful ablation was similar in patients treated with rhTSH compared to withdrawal [66, 67]. Pacini et al. [69], performed an study, with remnant ablation with 30 mCi (1110 MBq) after withdrawal of thyroid hormone, and after rhTSH aloneand after combining withdrawal and rhTSH, resulted in successful ablation in 84%, 54% percent, and 79% of patients, respectively. The rate was significantly lower in patients prepared with rhTSH alone. In another trial [70], remnant ablation with 54 mMi (2000 MBq) after withdrawal of thyroid hormone or after rhTSH resulted in similar rates of successful ablation. 

Although one retrospective study [71] found similar short-term recurrence rates in patients prepared with traditional thyroid hormone withdrawal, no long-term randomized study has been published comparing both approaches. 

Because of the lack of long-term data on recurrence and disease-specific survival, some centers are offering rhTSH ablation to only low risk patients preferring traditional hypothyroid withdrawal for patients at higher risk of recurrence or death from disease. This approach follows the American Thyroid Association guidelines [72]. 

The response of pulmonary metastasis to I131 is predicated on the size of the lesion, the age of the patient, and its ability to concentrate I131 [73, 74]. In contrast, bone metastases, which occur more commonly in follicular thyroid carcinoma, are less responsive to therapy. The use of recombinant human TSH (rhTSH) for remnant ablation and in the treatment of metastatic disease is now being studied and has the advantage of avoiding symptomatic hypothyroidism [75].

#### 2.2.6. Thyroid Hormone Suppression

Thyroid hormone suppression is routinely used after total thyroidectomy for differentiated thyroid cancer. Although randomized controlled trials are lacking, benefit was suggested in a large meta-analysis [76]. Higher risk patients are initially treated with a greater degree of suppression. Due to the risk of osteoporosis, many clinicians will now reduce the levothyroxine dose once the patient has been free of disease for several years.

#### 2.2.7. Role of External Beam Radiotherapy

Although prospective controlled trials are lacking, several studies have shown excellent local-regional control of disease with external beam radiotherapy (EBRT) in patients with locally advanced differentiated thyroid cancer after the primary surgery and before or after radioiodine [77–79]. Several groups have suggested that EBRT be considered in patients over 45 years with suspect persistent local disease after surgery which is unlikely to respond to radioiodine [78, 80].

#### 2.2.8. Diagnosis of Recurrent and Distant Disease

Recurrent well-differentiated thyroid cancer is not uncommon. Following total thyroidectomy, as many as 15% of patients will develop neck disease 0 to 5 years after initial therapy [64, 81]. Since the volume of recurrent or distant metastatic disease correlates relatively well with the prognosis, early diagnosis and management is essential. Whole body I131 scanning and should be performed in high risk patients 6 to 12 months after the primary surgery. Low risk patients can usually be evaluated by recombinant TSH (Thyrogen) stimulated thyroglobulin levels avoiding the need for an iodine scan. Since the majority of recurrences occur in the thyroid bed, neck ultrasonography represents the most sensitive means for detecting recurrence in the presence of elevated thyroglobulin. For those patients with elevated thyroglobulin and a negative iodine scan, PET/CT is exceptionally helpful in identifying recurrent and distant disease and has been shown to provide important prognostic information. Jadvar et al. evaluated 10 patients with suspected recurrent papillary thyroid cancer using PET and concluded in this small series that PET was useful in the evaluation of patients with suspected recurrent papillary thyroid cancer when the I131 scan is negative [82]. In a more recent study, eight patients underwent combined PET/CT scanning for suspected recurrence. Four (50%) of 8 patients underwent PET/CT indicating recurrence in the head and neck. A total of 11 lesions in these 4 patients were suspicious for recurrence on combined PET/CT imaging. Three patients with 8 lesions suspicious for recurrence on PET/CT underwent surgical removal of disease. All 3 patients had pathologic confirmation of recurrence, with 75.0% of the 8 lesions being positive. The authors concluded that combined PET/CT imaging is a valuable tool for the diagnosis and localization of recurrent thyroid cancer [83]. Recent evidence suggests that PET-positive disease is unlikely to I131 and may be associated with a more biologically aggressive tumor [84].

#### 2.2.9. Management of Recurrent Disease

The management of recurrent or metastatic disease largely depends on the extent of the disease. When there is microscopic persistent or recurrent disease, I131 dosing should be repeated. However when palpable gross tumor is identified, surgical management is the treatment of choice. When distant disease is identified, adjuvant I131 is usually the preferred. I131 scanning is the most accurate method for assessment of distant disease [85]. Noniodine avid tumors represent a challenge and may indicate a poorly differentiated tumor that is often associated with a more aggressive behavior and a poor prognosis [84]. EBRT may be used for gross residual disease unresponsive to radioiodine and not amenable to further surgery. External beam radiation or surgical resection can also be used for the management of pain or to decompress nerve root compression in the vertebrae however neither therapy has an impact on prognosis. There are few studies demonstrating the efficacy of systemic chemotherapy however there is data to suggest that doxorubicin may be effective in some patients (60–75 mg/m^2^ every 3 weeks) [86]. Currently, systemic chemotherapy is used only in clinical trials.

### 2.3. Hürthle Cell Carcinoma

Hürthle cell carcinoma (HCC) is a relatively rare form of thyroid cancer that is generally considered to be a more aggressive variant of follicular carcinoma. Similar to follicular carcinoma, the distinction between a benign Hürthle cell adenoma and a HCC is determined on the presence of vascular or capsular invasion. Similar to follicular cell carcinoma, the diagnosis cannot be made on fine needle aspiration or frozen section analysis. Patients with HCC tend to be older. In one review series, it was found that the median age of presentation for patients with follicular cell carcinoma was 55 years while the HCC patients presented at a mean age of 62 years. Patients presenting with HCC have a lower risk of regional metastasis at presentation when compared with follicular carcinoma, but a slightly increased risk of distant metastasis [63]. When followed long term, the number of patients that eventually develop distant metastasis is higher in HCC patients (30%) than papillary or follicular thyroid carcinoma patients. When distant metastasis occurs, 40% occur in the bone and as many as 30% occur in the lung [87].

#### 2.3.1. Management of Hürthle Cell Carcinoma

The management of HCC is not significantly different from follicular cell carcinoma. HCC with minimal capsular invasion (<1 mm) can be safely managed with a thyroid lobectomy and isthmusectomy. However, because it has been demonstrated that patients treated with a total thyroidectomy have a lower risk of recurrence [84, 88], recommend a total thyroidectomy. All high risk patients or patients with more extensive capsular invasion are managed with a total thyroidectomy. Following surgery, I131 is administered to achieve ablation and facilitate surveillance with thyroglobulin.

#### 2.3.2. Prognosis of Hürthle Cell Carcinoma

Less that 200 new cases of HCC carcinoma are diagnosed in the United States each year. As a result, there are few long term outcome studies however, Evans and Vassilopoulou-Sellin found in 1998 no difference in outcome between HCC and follicular cell carcinoma when cases are stratified by extent of invasion [89]. Like follicular cell carcinoma, the most important factor in assessing the prognosis seems to be the extent of capsular invasion. When tumors invade less than 1.0 mm, the rate of recurrence in exceedingly small, however as the extent of capsular invasion increases, the likelihood of regional and distant metastasis increases [89].

### 2.4. Medullary Thyroid Carcinoma

Medullary thyroid carcinoma (MTC) is derived from the nonepithelial parafollicular cells (c-cells) which produce the peptide calcitonin. Since the parafollicular cells embryo logically develop from the neural crest or diffuse neuroendocrine system, they commonly produce neuropeptides and catecholamines. Tumors deriving from diffuse neuroendocrine system, such as carcinoid tumors, pancreatic islet tumors, and pheochromocytomas, are cytologically and functionally similar. MTC exists in a sporadic and familial forms. While the gender distribution is roughly equivalent, the sporadic form of the disease occurs in 80 to 90% of newly diagnosed cases. The familial form of the disease occurs in an autosomal dominant trait as either isolated familial medullary thyroid carcinoma (FMTC), multiple endocrine neoplasia type IIA (MEN IIA), or multiple endocrine neoplasia type IIB (MEN IIB) (Table 5). Each of these familial forms of the disease is associated with a ret oncogene mutation located on chromosome 10. This provides a reliable screening tool to identify affected family members. Since the penetrance of medullary thyroid cancer is >90% in people with the RET gene mutation, prophylactic thyroidectomy is recommended at a young age [90].

#### 2.4.1. Clinical Presentation of MTC

Not unlike other forms of thyroid carcinoma, sporadic MTC typically presents as a thyroid nodule with or without an associated neck mass. Since MTC is derived from the diffuse neuroendocrine system, patients may present with a paraneoplastic syndrome resulting in diarrhea and abdominal cramping as a result of prostaglandin and vasoactive peptide release. The biological behavior and clinical presentation of MTC can be variable. Most tumors present as a well circumscribed encapsulated nodule within the thyroid gland. More aggressive tumors may present with a neck mass or in rare cases distant metastasis to the lungs, liver, or adrenal glands. In some cases, tumors may remain stable or quiescent while other tumors may progress rapidly. There has been little data to help predict the biological aggressiveness of MTC.

#### 2.4.2. Diagnosis of MTC

The most accurate method for diagnosis of MTC is an FNA of the thyroid nodule or neck mass. While cytodiagnostic features are recognized [91], immunostaining for calcitonin in the presence of negative thyroglobulin staining, is the most accurate method of diagnosis. When the calcitonin polymerizes, amyloid deposits can be found on histological analysis. There are no histological differences between the sporadic and familial forms of the disease. However, it is interesting to note that familial MTC has a predilection for presenting as a thyroid nodule at the junction of the upper third and lower two-thirds of the thyroid gland because this is the area of the gland in which the density of C cells is the highest. Sporadic disease usually presents as a unilateral nodule while familial disease is more commonly bilateral and multifocal. Familial disease is considered more aggressive and commonly presents in the second or third decades while sporadic disease presents later, in the sixth or seventh decades. 

*Newly diagnosed patients with MTC should be staged both biochemically and radiologically*. Measurements of serum *calcitonin* and carcinoembryonic antigen (CEA), to determine whether they are produced by the tumor and, if so, as a baseline for comparison with results obtained before and after surgery. Patients with higher preoperative serum calcitonin concentrations have larger tumors and are less likely to achieve normal concentrations after [92]. Patients who have normal serum CEA and serum calcitonin, are considered biochemically cured and have the best prognosiswith a five recurrence rate of only 5% [93]. 

The serum calcitonin concentration falls slowly in some patients, with the nadir not being reached for several months [94]. A high basal serum calcitonin value six or more months after surgery is presumptive evidence of residual disease. 

Subsequent follow-up should include periodic physical examination and measurements of serum calcitonin and CEA.

#### 2.4.3. Management of MTC

Medullary thyroid carcinoma requires a total thyroidectomy and paratracheal lymph node dissection. Larger thyroid tumors are associated with an increased risk of neck disease and therefore tumors greater than 2 cm should be treated with an ipsilateral selective neck dissection in addition to a bilateral paratracheal lymph node dissection [95]. Some have suggested that all patients should be treated with bilateral selective neck dissections because patients with unilateral intrathyroidal tumors, as many as 81% of patients have lymph node metastasis in levels II–V and 44% have disease in the contralateral lymph nodes level II–V [96]. Aggressive surgical therapy is warranted because occult metastases are common and surgery is the only effective method of therapy. There is no evidence that medical therapy has any role in the primary management of MTC and there is little evidence to suggest that adjuvant medical therapy will impact outcome. There is no role for radioiodine or thyroid hormone suppression in the treatment of medullary cancer. Brierley et al. treated a series of 40 patients at high risk for recurrence with external beam radiation and demonstrated an improvement in local-regional control (86% versus 52%) at 10 years [80, 97] however several other studies have failed to demonstrate an improvement in survival with external beam radiotherapy [98, 99]. Systemic chemotherapy has little impact on the natural course of the disease, and while Schlumberger et al. demonstrated 20% response rate in patients treated with doxorubicin, there is little data to support the use of systemic chemotherapy in MTC [100]. A new therapy using pretargeted anticarcinoembryonic antigen radioimmune therapy (RIT) has shown a survival benefit in a small clinical trial [101].

#### 2.4.4. Management of Regional Disease

When faced with regional recurrence, surgery is the treatment of choice. Regional metastasis mandates a neck dissection. Unlike well-differentiated thyroid cancer.

Radioactive iodine uptake is negligible in MTC and therefore aggressive surgical management of regional disease is justified. If a bilateral neck dissection was not performed at the time of initial therapy, bilateral neck dissections should be considered, even in the face of unilateral recurrence.

#### 2.4.5. Management of Distant Disease

Distant disease represents a significant dilemma. As discussed, conventional adjuvant therapy offers little for patients with regional or distant disease. Clinical trials are often offered at tertiary care medical centers.

#### 2.4.6. Management of Recurrent and Persistent Disease

When patients demonstrate a persistently elevated calcitonin level following surgery, it is because there is residual or recurrent disease. Identifying the location of the residual disease is often a challenge. CT, MRI, and PET have all been used with little success unless there is gross disease remaining in the neck or thyroid bed. The management of a patient with a persistently elevated calcitonin level and no radiographic evidence of disease is controversial. This is because there is evidence to suggest that this patient group does not necessarily have a compromised outcome [102] and when reoperation is pursued, the rate of success is low [103]. We recommend bilateral selective neck dissections for those patients that have not already undergone a neck dissection.

#### 2.4.7. Prognosis of MTC

Prognosis is better in younger patients, female gender, familial disease, and those with disease confined to the thyroid gland. The overall 5-year survival rate ranges from 25% to 75% [104, 105]. When nodal disease is present at the time of diagnosis, which occurs in 50% of patients, the 5-year survival rate drops to less than 50% [106]. A recent series suggests that the 5 and 10 year survival rates are as high as 90% and 80% [107].

### 2.5. Anaplastic Thyroid Carcinoma

Although less than 2% of all thyroid carcinomas are anaplastic thyroid carcinoma (ATC), it accounts for 14–39% of thyroid carcinoma deaths [108]. 

*Anaplastic Thyroid Cancers* are the most aggressive of the thyroid cancers. Mutations of the p53 tumor suppressor gene that lead to production of an inactive p53 protein occur in most anaplastic thyroid cancers, and not in other thyroid [109]. Mutations of the beta-catenin gene occur in up to 65 percent of anaplastic thyroid carcinomas [110]. It has been suggested that differentiated thyroid cancers may deteriorate into anaplastic thyroid cancers, it has been proposed that several different gene mutations provide an early growth advantage and that p53 mutations are the final step in the development of an anaplastic cancer. Evidence comes from studies describing well differentiated regions inside anaplastic cancers and the consequent mutation of BRAF, acquiring p53 mutation in a second step [111, 112]. However, in one study of 17 anaplastic cancers, no RET gene rearrangements were detected, providing no evidence for the evolution of papillary into anaplastic thyroid cancer [113]. 

Somatic mutations of the catalytic subunit of the phosphatidylinositol 3′-kinase (PIK3CA) have been identified in about 23 percent of anaplastic thyroid cancers [114]. In anaplastic thyroid cancers with well differentiated components, the PIK3CA mutations are restricted to the anaplastic component. 

The diagnosis of anaplastic thyroid carcinoma can be elusive. Not uncommonly, an FNA will be repeatedly interpreted as “undifferentiated carcinoma” until an open biopsy provides enough tissue to reveal the anaplastic nature of the carcinoma. Unlike other forms of thyroid cancer, anaplastic is characteristically aggressive and unrelenting. Accounting for less than 5% of malignant thyroid cancers, anaplastic thyroid cancer is often heralded by a rapidly growing thyroid mass with destruction of the adjacent cartilage, nerve and infiltrating muscles. 

The group of Chan et al. [115] in Hong Kong recently analyzed the clinico-pathological features, treatment and outcome of all patients with ATC treated over the past four decades in their institution. Fifty patients presenting with biopsy-proven ATC between 1966 and 2006 were studied. All patients were managed with surgery, radiotherapy, chemotherapy, or chemoradiation. Distant metastases at diagnosis were present in nine patients. The results confirmed the dismal outlook noted in previous studies, with a median survival of only 97 days, with 1- and 3-year survival of 14% and 8% respectively. Age 65 years or younger, lack of metastatic disease, surgical resection, and radiation to the neck were associated with better prognosis on univariate analysis. Cytotoxic chemotherapy was not associated with improved survival and disappointingly, no change in survival was seen over the 40-year period of the study. This and other studies emphasize the need for new treatment strategies for ATC [115–117].

#### 2.5.1. Clinical Presentation of ATC

Anaplastic thyroid carcinoma typically presents in the elderly population with a history of thyroid goiter [118]. Up to 20% of patients will have a history of well-differentiated thyroid cancer or co-existing well-differentiated thyroid cancer present within the thyroid specimen [119, 120]. Patients usually present with a rapidly growing neck mass, true vocal cord paralysis, and or dysphagia [118]. The mass tends to present as a fixed neck mass invading the surrounding structures. Direct invasion of the airway and larynx can lead to hoarseness or airway obstruction. As the lesion becomes more advanced, esophageal invasion can result in dysphagia. Anaplastic carcinoma may arise de novo or as a “conversion tumor” arising in the presence of a preexisting papillary or follicular carcinoma. When anaplastic carcinoma arises de novo, the tumor may progress rapidly metastasizing to the lungs, liver, and bones within weeks of presentation.

#### 2.5.2. Diagnosis of ATC

A FNA is often the first approach to diagnosis however necrosis and degeneration of the tumor may occur as the tumor rapidly out grows its blood supply. A FNA aspiration may yield necrotic material. Alternatively, the FNA may yield poorly differentiated cells suggestive of either lymphoma, anaplastic thyroid cancer, or poorly differentiated carcinoma of unknown origin. Flow cytometry is useful to rule out lymphoma, however immunohistochemistry and electron microscopy may be necessary to confirm the diagnosis of anaplastic thyroid carcinoma.

#### 2.5.3. Management of ATC

There is controversy regarding the ideal management of anaplastic carcinoma largely because it is rapidly progressing and poorly responsive to therapy [121]. Defining the goals of management is essential. The options for therapy range from multimodal therapy, surgical resection, debulking, radiotherapy, chemotherapy, and palliative therapy [118, 122, 123]. Early disease, disease confined to the thyroid gland, is poorly responsive to unimodality therapy, however in a recent study, investigators reviewed a cohort of 516 patients with anaplastic thyroid carcinoma reported to 12 population based cancer registries between 1973 and 2000. They found that patients less than 60 years old with intrathyroidal anaplastic thyroid cancer appear to have a better prognosis than older patients with extra-thyroidal spread following total thyroidectomy and external beam radiation [124]. Long term survivors are few however in those that do survive the disease, they commonly have only a small focus of anaplastic tumor arising within a preexisting well-differentiated thyroid cancer [17, 125]. Adjuvant therapy has also been controversial. Standard fractionated radiation therapy has not been demonstrated to significantly change the clinical course [126]. Hyperfractionated therapy has resulted in a marginal improvement in response rates however the side effects, including esophagitis, can be debilitating [127]. Tennvall et al. reported using preoperative hyper fractionated radiation (30 Gy), doxyrubicin, and surgical resection followed by postoperative radiation (46 Gy). They demonstrated promising results with this regimen, controlling local recurrence in 48% of patients and disease free survival in 12% [128]. In advanced disease, the goals include establishing an airway, a source of nutrition, and an acceptable quality of life. Surgery is warranted for the control or prevention of airway obstruction or extensive skin ulceration.

#### 2.5.4. Prognosis of ATC

Anaplastic thyroid carcinoma is one of the most aggressive human malignancies. It is associated with an almost uniformly rapid and lethal clinical course [129]. Several studies have confirmed the universal lethal course associated with anaplastic thyroid cancer. Patient age, gender, tumor size, extent of disease, leukocytosis, presence of acute local symptoms, coexisting multinodular goiter and well differentiated thyroid carcinoma, surgical resection, and multimodal therapy all reportedly influence patient survival according to some studies [130, 131]. However, there are few interventions that have improved survival.

## 3. The New Insights

### 3.1. New Agents in the Treatment of Differentiated Thyroid Cell Carcinoma

*Axitinib (AG-013736)* is a potent small molecule inhibitor of VEGF receptors 1, 2, and 3. A single arm multicenter study evaluated the efficacy of this novel compound in advanced thyroid cancers. Sixty patients with metastatic or unresectable locally advanced thyroid cancer refractory to or not suitable candidates for radioiodine treatment, received axitinib at a starting dose of 5mg orally twice daily. The majority of the patients had differentiated thyroid cancer and two had anaplastic cancer. There were 18 partial responses and 30 patients had stable disease. The median progression free survival had not been reached at a median follow up of 273 days. Therapy was well tolerated with fatigue being the main side effect. Importantly the study was a proof of principle as levels of soluble VEGF receptor were consistently decreased and levels of VEGF in the blood were increased demonstrating pharmacodynamic activity against targeted VEGF receptors [132, 133]. 

*Sorafenib*, a multitargeted small molecule kinase inhibitor, including the VEGF receptor and BRAF kinase, has also been evaluated in patients with thyroid cancer. Of 19 evaluable patients with metastatic, iodine refractory, papillary thyroid cancer enrolled in a phase II trial of sorafenib, five patients achieved a partial response and eight had stable disease with sorafenib. Median response duration was 14.2 months (12–15þ months) and median time to progression of all patients was 4þ months (4 days to 14.5þ months). Pharmacodymanic analysis of tumor tissue before and after sorafenib treatment (at 1 and 2 weeks) in two patients revealed reduction in pERK (downstream of VEGFR and BRAF) and pAKT (downstream of VEGFR) [134]. Sorafenib is currently US Food and Drug Administration approved for the treatment of renal cell cancer and hepatocellular carcinoma. It has side effects including a desquamating rash often on the hands and can cause gastrointestinal symptoms. It is currently being used in many trials across the world and in many malignancies and its role in the treatment of thyroid cancer is likely to increase. 

In the Phase II trial of Gupta-Abramson et al. [135], thirty patients were treated for a minimum of 16 weeks with Sorafenib 400 mg twice daily. Seven patients had a partial response lasting from 18 to 84 weeks. Sixteen patients had stable disease lasting from 14 to 89 weeks. Seventeen (95%) of 19 patients for whom serial thyroglobulin levels were available showed a marked and rapid response in thyroglobulin levels with a mean decrease of 70%. The median PFS was 79 weeks. Toxicity was consistent with other sorafenib trials, although a single patient died of liver failure that was likely treatment related. 

The overall clinical benefit rate (partial response stable disease) in this study was of of 77%, median PFS of 79 weeks, and an overall acceptable safety profile. 

*AMG-706* Finally, in a third abstract reported at the 2007 annual meeting of the American Society of Clinical Oncology, AMG-706, another multikinase inhibitor (VEGF/PDGF receptors, Kit and RET), was studied in a phase II trial of patients with advanced differentiated thyroid cancer or medullary thyroid cancer. All subjects received AMG 706 daily until disease progression or unacceptable toxicity. The results for the differentiated group with median follow up of 32 weeks were presented at the meeting. Twelve percent of patients had a partial response and another 69% had stable disease. Side effects were tolerable with diarrhea and hypertension being the most common severe toxicities (11 and 22%, resp.) [136]. These three studies show the renewed interest in refractory thyroid cancer management and that small molecule inhibitors offer one of the most exciting prospects for the treatment of these patients. 

*Cyclo-oxygenase 2 (COX-2)* is an enzyme overexpressed in many thyroid cancers. Inhibition of this enzyme has been shown to reduce progression of tumors in animal models and humans. Two common oncogenes in papillary thyroid cancer, RET/PTC1 and RET/PTC 2, induce expression of COX2. Celecoxib, a widely available selective COX-2 inhibitor, seemed therefore to be an attractive potential treatment for this disease. Unfortunately an early study using this drug failed to show a benefit at a dose of 400 mg twice daily [137]. 

*Gefitinib (Iressa)* is a tyrosine kinase inhibitor of the epidermal growth factor receptor (EGFR). This receptor is highly expressed on normal and malignant thyroid tissue, and has been associated with a worse prognosis in well differentiated thyroid cancer. Preclinical studies have established in-vitro activity of gefitinib against thyroid cancer. Although a phase II clinical trial with gefitinib reported no objective responses by RECIST criteria in 27 patients with radioiodine resistant thyroid cancer, reduction in tumor volume was noted in 32% of the patients, and 12% of patients had no evidence of progression after one year of treatment. Thyroglobulin levels were decreased to 90% of baseline in five patients with stable disease. Overall survival was 17.5 months in the cohort [138, 139].

### 3.2. New Agents in ATC

Some of the multikinase inhibitors mentioned above have shown hints of activity in the few patients enrolled with ATC. Another antiangiogenesis compound, combrestatin A4 phosphate (CA4P), has further been evaluated in a cohort of patients with ATC. Unlike the other angiogenesis inhibitors such as bevacizumab, an antibody against VEGF, or the VEGF receptor kinase inhibitors, CA4P is a tubulin-binding vascular disrupting agent that stops blood flow through existing blood vessels, depriving the tumor of its nutrients and oxygen. A phase II study with this agent in 18 patients with ATC was reported last year. Although there were no objective responses, six patients had stable disease and 28% had no progression of disease for greater than 3 months [28, 140]. Based on these results, an international phase III study has been planned evaluating the use of CA4P in combination with the widely used chemotherapy pair of paclitaxel and carboplatin. Patients with unresectable ATC will be randomized to chemotherapy with or without CA4P. The study is a huge undertaking given the rare and aggressive nature of the disease, and offers some hope to a population of patients with no other effective treatments.

### 3.3. New Agents in MTC

**Figure 1:**
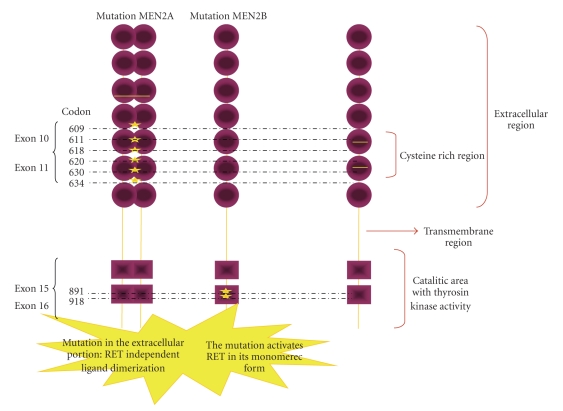
RET mutations in medullary thyroid carcinoma.

One of the hallmarks of MTC is the association with the rearranged during transfection protooncogene (RET) (Figure 1). In normal states, this gene is involved in cell signaling, and regulates the production of proteins that are essential for spermatogenesis and the development of the autonomic nervous system and kidneys. RET is a transmembrane protein that can be activated by extracellular signals and initiate a complex cascade of intracellular reactions regulating cell growth. A tyrosine kinase enzyme located on the intracellular domain of RET activates this cell signaling cascade. Mutations in specific regions of the RET gene have been described in MTC, and these occur in both the sporadic and familial form of the disease [141, 142]. These mutations have led to fascinating experiments looking at corrective gene therapy as a treatment strategy. ZD6474 (Zactima, vandetanib) is a novel anilinoquinazoline compound with a molecular weight of 475 Daltons. It was considered worthy of further development after demonstrating potent inhibition of VEGFR-2 tyrosine kinase in recombinant enzyme assays as well as additional activity against VEGFR-3, EGFR and RET tyrosine kinases. Vandetanib showed excellent selectivity for these kinases compared with related receptor tyrosine kinases such as platelet-derived growth factor receptor (PDGFR)-b and c-Kit [143–145]. Its activity in hereditary MTC has been established in a single arm phase II study, which reported a 17% confirmed and 7% unconfirmed partial response rate in 30 patients, with a 50% stable disease rate. In 23 patients there was a decrease in calcitonin levels by at least 50% which was maintained for 4 weeks or longer. Toxicities were manageable, with the most common adverse events being diarrhea, rash and asymptomatic QTc prolongation. The encouraging results of this trial have spurred accrual onto an ongoing international randomized phase II trial comparing ZD6474 to placebo [146]. Once the results from this trial are available, the authors feel that there will probably be enough evidence for the approval of ZD6474 in the treatment of metastatic or unresectable MTC, a true landmark in this disease.

## Figures and Tables

**Table 1:** Risk factors for thyroid carcinoma.

History of radiation exposure
Family history of papillary thyroid carcinoma
Single dominant solid nodule greater than 4 cm
Male gender
Rapid growth of a nodule
Younger than 20 years old
Older than 70 years old
Cervical metastasis
Evidence of invasion on imaging

**Table 2:** AGES classification system.

Prognostic score = 0.05 × age (if age !40)
+1 (if grade 2)
+3 (if grade 3 or 4)
+1 (if extrathyroidal)
+3 (if distant spread)
+0.2 × tumor size (cm maximum diameter)
Survival by AGES score
3.99 = 99%
4–4.99 = 80%
5–5.99 = 67%
5–6 = 13%

**Table 3:** AMES classification system.

Low Risk
Young patients (men “41 years old, women” 51 years old)
without distant metastasis
Older patients (intrathyroidal papillary thyroid cancer,
minor capsular invasion for follicular lesion)
Primary cancers <5 cm in diameter
No distant metastasis
High Risk
All patients with distant metastasis
Extrathyroidal papillary
Major capsular invasion for follicular
All older patients with extrathyroidal spread
All older patients with primary cancer >5 cm in diameter
(men > 40, women > 50)
Survival by AMES score
Low risk = 99%
High risk = 61%

**Table 4:** TNM classification system for thyroid carcinoma.

TNM Classification System
T1 Tumor diameter 2 cm or smaller
T2 Primary tumor diameter > 2 to 4 cm.
T3
Primary tumor diameter > 4 cm limited to the thyroid or with
minimal extracapsular
extension
T4a
Tumor of any size extending beyond the thyroid capsule to
invade the subcutaneous soft
tissues, larynx, trachea, esophagus, or recurrent laryngeal nerve
T4b Tumor invades prevertebral fascia or encases carotid artery
or mediastinal nerves
Tx Primary tumor size unknown, but without extrathyroid
extension
N0 No metastatic nodes
N1a Metastasis to level VI (pretracheal, paratracheal, prelaryngeal)
NIb Metastasis to unilateral or bilateral or contralateralcervical
or superior mediastinum
Nx Nodes not assessed at surgery
M0 No distant metastasis
M1 Distant metastasis

**Table 5:** Familial medullary thyroid carcinoma.

*Familial Medullary Thyroid Cancer*
MEN IIA
Medullary thyroid cancer
Parathyroid adenoma
Pheochromocytoma
MEN IIB
Medullary thyroid carcinoma
Pheochromocytoma
Ganglioneuromatosis
Marfanoid habitus
